# Economic impact of a peste des petits ruminants outbreak and vaccination cost in northwest Ethiopia

**DOI:** 10.1111/tbed.14544

**Published:** 2022-04-12

**Authors:** Wudu T. Jemberu, Theodore J. D. Knight‐Jones, Alemseged Gebru, Sefinew A. Mekonnen, Andnet Yirga, Demeke Sibhatu, Jonathan Rushton

**Affiliations:** ^1^ Department of Veterinary Epidemiology and Public Health University of Gondar Gondar Ethiopia; ^2^ International Livestock Research Institute Addis Ababa Ethiopia; ^3^ Department of Veterinary Science Bahir Dar University Bahir Dar Ethiopia; ^4^ National Animal Health Diagnostic and Investigation Center Sebeta Ethiopia; ^5^ Institute of Infection and Global Health University of Liverpool Liverpool UK

**Keywords:** economic, Ethiopia, outbreak, peste des petits ruminants, small ruminants, vaccination cost

## Abstract

Peste des petits ruminants (PPR) is an important endemic disease of small ruminants in Ethiopia. While vaccination is widely used in the country to control the disease, quantitative estimates of the actual economic losses due to outbreaks and costs of vaccination are scarce. This study assessed the economic impact and costs of PPR vaccination in Metema district, northwest Ethiopia. The economic impact of the disease was estimated from an outbreak investigation including interviews with 233 smallholder farmers in PPR‐affected kebeles (subdistricts). The cost of PPR vaccination was obtained from vaccination programs in six kebeles of the district and from secondary data in the district veterinary office. In the investigated PPR outbreak, animal‐level PPR morbidity and mortality rates were 51% and 22%, respectively, in sheep and 51% and 25%, respectively, in goats. The flock level morbidity rate was 83% for sheep flocks and 87% for goat flocks. The mean flock level loss was Ethiopian Birr (ETB) 7835 (USD 329 in 2018 average exchange rate) (95% CI: 5954‐9718) for affected sheep flocks and ETB 7136 (USD 300) (95% CI: 5869–8404) for affected goat flocks. The losses in all study flocks during the outbreak were ETB 319 (USD 13.4) per sheep and ETB 306 (USD 12.9) per goat. Mortality accounted for more than 70% of the total losses in both sheep and goat flocks. Vaccination costs for PPR were estimated at ETB 3 per correctly vaccinated animal. Based on the estimated animal‐level direct economic losses and vaccination cost, it can be conjectured that vaccination will pay if a district PPR outbreak occurs more than once every 13 years. This does not account for additional benefits from vaccine‐derived herd immunity reducing disease burden in the wider population. In conclusion, PPR caused high morbidity and mortality in the affected flocks and resulted in high economic losses, equivalent to 14% of annual household income, dramatically affecting the livelihoods of affected flock owners. The vaccination practised in the district is likely to have a positive economic return, with strengthened vaccination programmes bringing reduced economic impact and improved livelihoods.

## 1 INTRODUCTION

Peste des petits ruminants (PPR) is an acute or subacute, highly contagious, and economically important transboundary viral disease that imposes a significant constraint upon sheep and goat production in Africa and Asia. It is a frequently fatal disease of sheep and goats caused by the PPR virus (PPRV) of the *Morbillivirus* genus and *Paramyxoviridae* family (OIE, 2020). The disease may affect up to 100% of animals in the flock, and an outbreak can kill between 20% and 90% of exposed animals (Albina et al., 2013; Hegde et al., 2009). The disease leads to significant economic, food security, and livelihood impacts in affected communities. In addition to severe production losses associated with mortality and morbidity and costs of control, PPR can also limit trade and prevent the development of intensive small ruminant production (Singh et al., 2014).

Ethiopia has a substantial small ruminant population estimated at 31.3 million sheep and 32.7 million goats (Central Statistical Agency [CSA], 2018). Small ruminant production in the country has been burdened with several high‐impact transboundary diseases, such as PPR, sheep and goat pox, and contagious caprine pleuropneumonia, with PPR considered a priority livestock disease in Ethiopia (Magona et al., 2016; Waret‐Szkuta et al., 2008). For instance, a 5‐year (2010−2014) retrospective study revealed a high PPR incidence in the Amhara region (containing 29% of Ethiopian small ruminant population) with 63 reported outbreaks (Fentie et al., 2018).

Vaccination plays a key role in controlling many animal diseases, including PPR, for which a highly effective vaccine is widely used (Roth, 2011; Shimshony & Economides, 2006). It is important to understand the economic justification for any animal health program, including PPR, which is publicly funded in Ethiopia, with many districts conducting vaccination. However, there is a lack of empirical data on the economic impact of the disease and the cost‐effectiveness of PPR vaccination. The aim of this study was to estimate the economic impact of PPR outbreaks on smallholder farmers and the costs of vaccination, looking at the case of Metema district in northwest Ethiopia.

## 2 MATERIALS AND METHODS

### 2.1 Description of the study area and study population

**FIGURE 1:**
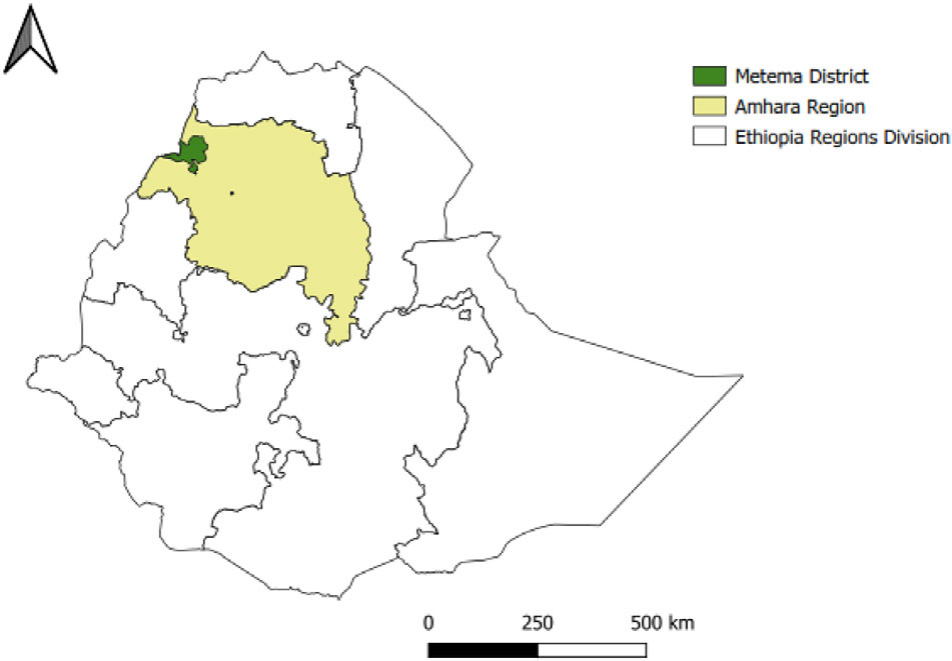
Map of the study area showing Metema district, Ethiopia

The study was conducted in the Metema district of Amhara Regional State in northwest Ethiopia (Figure 1). Metema district was selected for this study because it has a substantial small ruminant population (152 488 sheep and goats; CSA, 2018), is frequently affected by PPR outbreaks and annual PPR vaccination is practised, albeit with irregular coverage (Yirga et al., 2020). The district has 24 kebeles (subdistricts) and covers an area of approximately 7000 km^2^. The small ruminant flocks have sheep, goats, or both species. The flocks are kept under extensive grazing systems where flocks from different households mix when grazing and at watering points. Sheep and goats in the district are kept mainly for income generation from the sale of live animals and for home meat consumption but not for milk production (Gizaw et al., 2010).

### 2.2 Sampling and data collection

#### 2.2.1 Outbreak impact data

Data for estimating morbidity and mortality rates and associated economic impacts were collected from smallholder farmers in kebeles (subdistricts) affected by a PPR outbreak that occurred from the beginning of September 2017 to the end of January 2018. Data for estimation of vaccination cost were collected from other kebeles in the district where preventive vaccination was conducted during the same period. Kebeles with a suspected PPR outbreak were identified from the district veterinary office, and the outbreak was confirmed by collecting clinical samples as described in Section 2.3.

A total of 233 farmers from the six kebeles affected by the outbreak were enrolled in the study. The farmers were selected from different community gathering sites, such as community meetings, churches, and markets. Farmers in these gathering sites were approached, and those willing were interviewed irrespective of the outbreak status of their flocks. Economic impact data were collected using a structured questionnaire (Appendix 1) that was administered to the farmers during face‐to‐face interviews conducted in the local language (Amharic) by the researchers at the end of the outbreak. The questionnaire was designed primarily to record household flock morbidity, mortality, and production losses. Small ruminants were categorized according to their production status. The five categories were adult nonpregnant females, adult pregnant females, adult males, young females and males (3 months–1 year old), and lambs and kids (younger than 3 months). Adult females were differentiated as pregnant and nonpregnant based on farmer‐observed signs of pregnancy; this was used to identify cases of PPR‐induced abortions.

#### 2.2.2 Vaccination data

Vaccination data were collected from six kebeles conducting PPR vaccination during the study period and from secondary data in the district veterinary office. Data were collected for 11,066 sheep and goats that were presented for vaccination at different vaccination sites visited in the six kebeles.

Informed oral consent was obtained from each participating flock owner after reading a written explanation of the purpose of the study, the risks and benefits of participation in the study, their right to refuse to participate in the study, and the conditions of confidentiality. Animals were handled humanely during sample collection. This study was approved both for human and animal research ethics by the Institutional Review Board of the University of Gondar.

### 2.3 PPR outbreak confirmation

PPR was first suspected by district animal health personnel based on clinical and epidemiological characteristics. Clinical samples were then collected from two affected kebeles by the research team. A total of 20 swab samples (nasal, ocular, and oral and/or gum debris) collected from 12 diseased animals in four flocks were assessed for the presence of PPRV antigen.

Swab samples were collected using sterile swabs that were placed in a transport medium containing phosphate buffer solution (PBS) before being taken to the National Animal Health Diagnostic and Investigation Center in Sebeta, Ethiopia. Here, samples were subjected to immuno‐capture enzyme‐linked immunosorbent assay (ELISA) using the ID Screen® PPR Sandwich ELISA test kit (IDVet, CIRAD‐EMVT, Montpellier, France) to detect PPRV antigen. The optical density value for each sample, which correlates with the quantity of PPR antigen in the sample, was converted to percentage positivity, and samples with values ≥20% were considered positive for PPRV antigen (Pourquier et al., 2013).

### 2.4 Morbidity and mortality data

During the investigation, to confirm whether a selected flock was affected by PPR during the outbreak, farmers were asked if any disease outbreak had occurred in their flock in the preceding 3 months and if so, they were asked to explain the main epidemiological and clinical features of the outbreak. If the farmer's description matched recognized characteristics of PPR (mucopurulent ocular and/or nasal discharge, oral lesions and/or diarrhoea, which appeared to be contagious affecting several animals in flock), it was considered that PPR had occurred in the flock.

Then, for each production category of sheep and goats, the farmer was asked how many animals were at risk and how many experienced clinical disease, abortion (in case of pregnant animals), or death. PPR treatment costs by group were also ascertained. The animal‐level morbidity rate was estimated as the number of animals clinically diseased divided by the total number of animals at risk (considering all sheep and goats in all flocks included in the study), and the flock‐level morbidity rate was determined as the number of positive flocks (flocks with one or more animals with clinical PPR) divided by the total number of flocks at risk, that is, all flocks included in the study. The mortality rate was determined as the number of animals that died of PPR during the outbreak divided by the total number of animals at risk.

### 2.5 Estimation of economic impacts

#### 2.5.1 Mortality loss

Financial loss due to mortality was calculated by considering the five production categories of sheep and goats that died and their corresponding market prices. Price information was collected from four primary markets within the study area by asking the price of 5–10 sheep and goats for each category. All monetary data were collected and calculated using ETB, where 1 ETB was equivalent to USD 0.042 using the 2018 average exchange rate.

Economic loss due to mortality in a flock was calculated as:

(1) ML=ΣNADj∗Pj

where ML represents the mortality losses due to PPR in a flock; NADj is the number of animals that died in category j; and Pj is the average price of the animals that died in category j, where j represents the different production categories from j_1_ to j_5_.

#### 2.5.2 Body weight loss

Sheep and goats that survive clinical PPR lose weight, and their market value decreases due to this weight loss. The economic loss due to bodyweight loss from PPR was estimated by comparing weights (measured by weight balance) between PPR‐affected and nonaffected sheep and goats of similar age and sex in the same flock. When nonaffected animals of similar age and sex were not available in the same flock, animals from a neighbouring flock were used. Four to six pairs of animals were weighed for each category of sheep and goats from all study flocks for this purpose. The difference in weight between affected and unaffected animals was considered the weight loss due to the illness. The economic loss due to body weight loss was estimated as follows:

(2) WL=ΣRAj∗BWLj∗PL

where WL represents the economic loss due to PPR‐induced body weight loss in a flock, RAj is the number of PPR recovered animals for category j, BWLj is the average estimated body weight loss for category j, and PL is the average price of live weight/kg.

#### 2.5.3 Abortion loss

The loss due to PPR‐induced abortion that was noticed by farmers was estimated. The loss of abortion was difficult to estimate in monetary terms, and the cost of a single aborted fetus was assumed to be half the price of a newborn lamb/kid. Abortion loss was estimated using the following formula:

(3) AL=Nabf∗Pn

where AL represents the abortion loss in the infected flock, Nabf is the number of foetuses aborted due to PPR in the flock, and Pn is the financial loss from an abortion, which was assumed to be equivalent to 150 ETB (half of the estimated price if new a born lamb/kid).

#### 2.5.4 PPR treatment cost

Treatment cost represents the expense incurred by farmers for the diagnosis and treatment of clinically sick animals at the local public veterinary clinic. The labour cost that farmers incurred for the treatment of sick animals was also included in the treatment cost. Flock‐level treatment cost data were collected by asking farmers. The cost of PPR treatment was calculated as:

(4) $$\mathrm{TC}=\mathrm{DMC}+\left(\mathrm{NhoursL}*\mathrm{Prl}\right)$$

where TC represents the treatment cost for an affected flock; DMC is diagnosis and medication cost for a flock; NhoursL is the average reported number of working hours the farmer lost from treating sick animals, and Prl is the average price of replacement labour per hour (which was ETB 10/h, derived from the ETB 80 daily wage for unskilled labour).

#### 2.5.5 Overall economic losses

The overall economic loss per individual flock was obtained by adding mortality loss, body weight loss, abortion loss, and treatment cost:

(5) OEL=ML+WL+AL+TC

where OEL represents the overall economic loss due to PPR per affected flock.

### 2.6 Estimation of the cost of PPR vaccination

Vaccination in the district was carried out by the district veterinary office. The district veterinary office buys the vaccine from the manufacturer, transports it to the district, and stores it under cold chain conditions until use in the field. Vaccination campaigns are organized by the district veterinary office, and personnel from the district office are deployed to the field vaccination sites to conduct vaccination.

When calculating the operational cost of PPR vaccination, we accounted for vaccine price, field vaccine delivery cost, vaccine transport cost, vaccination mobilization and coordination cost, the number of sheep and goats vaccinated per person (animal health personnel)/day, proportion of vaccine wastage, and amount of time spent by each farmer to get his flock vaccinated. Data on vaccine prices, vaccine transport cost, and vaccination mobilization and coordination costs were collected from the veterinary office of the district. Data on the number of sheep and goats vaccinated per person/day, vaccine wastage, and amount of time spent by each farmer to get his/her animals vaccinated were obtained by observation and asking farmers during PPR vaccination.

#### 2.6.1 Cost components

Vaccination cost components were computed according to the following formulae adapted from Lyons et al. (2019):

(6) VC=Vc+Vtc+Fdc+Cc+Ftc

where VC represents the vaccination cost; Vc is the vaccine cost; Vtc is the vaccine transport cost; Fdc is the field vaccine delivery cost; Cc is the coordination cost; and Ftc is the cost of farmers’ time lost to get their animals vaccinated.

Where:

(7) Vc=Vp+Sp

Vc represents the vaccine cost; Vp is the vaccine price, which was ETB 0.49/dose, and Sp is the saline price used for reconstituting the vaccine, which was ETB0.04/dose.

(8) Vtc=Tc+Fc+Pc

Vtc represents the vaccine transport cost after purchase; Tc is the transport truck rental cost; Fc is the fuel cost; and Pc is the cost of transporting personnel.

(9) Fdc=Cp+Tc+Mp

Fdc represents the field vaccine delivery cost; Cp is the cost of field vaccination personnel (per diem); Tc is the cost of field transport (car rent, fuel, and car maintenance cost); and Mp is the price of materials (consumables).

(10) $$\mathrm{Cc}=\left(\mathrm{Cd}*\mathrm{Pd}\right)+\mathrm{Tc}$$

Cc represents the coordination cost, Cd is the number of coordination days, Pd is per diem, and Tc is the transport cost of the local vaccination coordinator.

(11) Ftc=Fh∗Wh

Ft represents farmers’ time lost during vaccination; Fh represents hours spent by the farmers in getting their flocks vaccinated, and Wh is farmers’ wage, which was ETB 10/h.

#### 2.6.2 Vaccination wastage

During direct observation of vaccination, the quantity of vaccine doses wasted was recorded, including wastage from doses not given correctly that needed to be repeated, vaccine spilt during removal of air from syringes, and vaccine discarded having exceeded the maximum time (30 minutes) between reconstitution and use. This wastage was measured by close monitoring of injections given from the beginning to the end of a full vaccination syringe (typically 30 doses). By measuring the number of doses wasted, the number of doses correctly administered (correctly vaccinated animals) was calculated from the total number of doses distributed. Then, each component cost of vaccination was calculated per correctly vaccinated animal.

## 3 RESULTS

### 3.1 Small ruminant flock size and structure

A total of 233 smallholder farmers were surveyed in six PPR outbreak‐affected kebeles, of which 130 had goat flocks, 81 had sheep flocks, and 22 had mixed flocks. The flock size and structure of the studied small ruminant flocks are presented in Table 1. The average flock size was approximately 20 for both sheep and goat flocks. The flock size for the 22 mixed (sheep and goat) flocks was larger, with an average flock size of >100 animals (sheep and goats combined). The mixed flocks were excluded from further analysis, as it was difficult to obtain detailed individual animal morbidity data from owners due to the large number of animals in the flock.

**TABLE 1:** Small ruminant flock size and structure

**Flock structure**		Goat flock	Sheep flock
Number of flocks		130	81
Average flock size (interquartile range)		20.25 (10‐25)	20.32 (10‐25)
Flock structure	Adult female nonpregnant (%)	26.9	27.8
	Adult female pregnant (%)	12.6	11.2
	Adult male (%)	3.8	3.4
	Young female and male (3‐12 months inclusive) (%)	25. 7	25.2
	Kids and lambs (below 3 months of age) (%)	31.0	32.4

### 3.2 Outbreak confirmation

Out of 20 samples collected from conjunctival swabs, nasal swabs, and buccal debris of 12 PPR suspected animals, 14 (70%) samples from 8 animals (75%) were positive for PPR viral antigen by Ic‐ELISA. The 8 positive animals were found in all four flocks and two kebeles sampled. Based on this result, the outbreak occurring in the affected kebeles was confirmed to be due to PPRV.

### 3.3 Morbidity and mortality

Of 130 goat flocks and 81 sheep flocks enrolled in the study, 113 and 67 were affected by PPR, giving a flock level morbidity rate of 87% and 83% in goats and sheep flocks, respectively. Based on farmers’ diagnosis, morbidity and mortality rates in different categories of sheep and goats in the study flocks (note that study flocks included both outbreak affected and unaffected flocks) are presented in Table 2.

**TABLE 2:** PPR morbidity and mortality in different categories of sheep and goats in the study flocks (note that study flocks including both outbreaks affected unaffected flocks)

Species	Category	Total No. of animals	Morbidity (% [95%CI])	Mortality (%[95%CI])
Goat	Nonpregnant	707	41.3 (37.6‐45.0)	16.1 (13.8‐15.0)
	Pregnant	339	58.7 (53.2‐63.0)	12.0 (8.8‐16.0)
	Adult male	99	21.2 (13.6‐30.6)	7.0 (2.9‐14.0)
	Young female and male	677	56.2(52.6‐60.0)	31.6 (28.1‐35.3)
	Kids	811	56.4 (52.8‐59.8)	35.1(32.0‐38.8)
	Overall	2633	51.3 (49.4‐53.2)	25.1 (23.5‐26.8)
Sheep	Nonpregnant	456	43.0 (38.4‐47.7)	18.7 (15.2‐22.5)
	Pregnant	185	59.4 (52.0‐66.6)	9.7 (5.9‐14.9)
	Adult male	56	23.2 (13.0‐36.4)	10.7 (4.0‐21.9)
	Young female and male	415	53.7 (48.8‐58.6)	25.3 (21.2‐29.8)
	Lambs	534	54.7 (50.3‐59.0)	26.4 (22.7‐30.4)
	Overall	1646	50.7 (48.3‐53.2)	21.6 (19.7‐23.7)

The overall morbidity and mortality rates across the study flocks were 50.7% and 21.6%, respectively, in sheep and 51.3% and 25.1%, respectively, in goats. In the affected flocks, the morbidity and mortality rates were 56% and 24%, respectively, in sheep and 56% and 27%, respectively, in goats. Morbidity and mortality rates were highest in lambs and kids and lowest in adult males in both species.

### 3.4 Economic impacts of PPR

**FIGURE 2:**
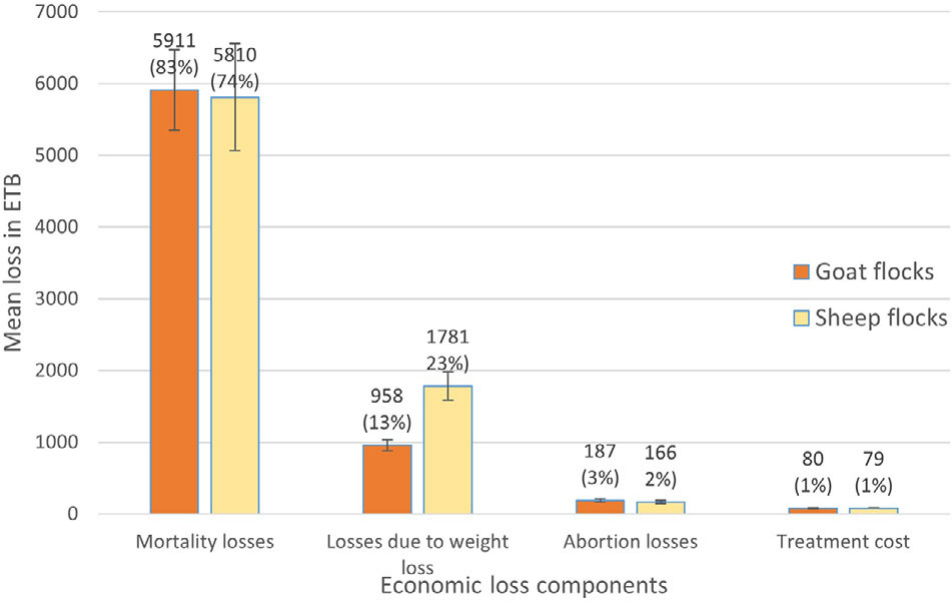
The components of mean economic losses per affected flock due to PPR with standard error bars

The median flock‐level economic losses for the study flocks were ETB 4354 (USD 182.9) in sheep and ETB 4765 (USD 199.3) in goat flocks, and the mean animal‐level losses were ETB 319 (USD 13.4)/sheep and ETB 306 (USD 12.9)/goat. The detailed flock‐level losses for affected flocks are shown in Figure 2. The mean flock level losses were ETB 7835 (USD 329.1) (95% CI: ETB 5954 – 9718) for affected sheep flocks and ETB 7136 (USD 299.7) (95% CI: ETB 5869–8404) for affected goat flocks. The interquartile ranges for these estimates were ETB 2988 −10275 for affected sheep flocks and ETB 3010‐8859 for affected goat flocks. Mortality was the major loss component accounting for more than 70% of losses followed by weight loss due to PPR morbidity in both sheep and goat flocks. Weight loss contributes more to sheep flocks than goat flocks (Figure 2).

### 3.5 Costs of PPR vaccination

**FIGURE 3:**
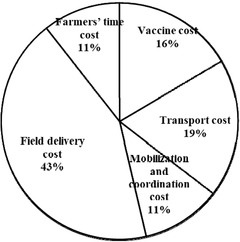
Components of PPR vaccination costs

The cost per dose of correctly vaccinated animals was estimated to be ETB 3.00 (USD 0.13). This cost was composed of vaccine cost, including reconstituted saline (ETB 0.50), field delivery cost (ETB 1.3), vaccination transportation (ETB 0.57), vaccination coordination cost (ETB 0.33), and opportunity cost of farmers’ time (ETB 0.32). The percentage of vaccine doses wasted was found on average to be 22% and was used to adjust the total doses distributed to calculate the number of doses correctly administered or correctly vaccinated animals. The major component of the vaccination cost was field delivery cost, which accounted for 43.6% of the total cost per effectively vaccinated animal (Figure 3).

## 4 DISCUSSION

In this study, we observed a very high household economic impact for flocks affected by PPR (USD 300‐329/affected flock approximately 13%–14% annual household income of smallholders in Ethiopia). At the same time, vaccination costs were low (USD 0.13/animal) and could be even lower with less vaccine wastage (> 20% wasted). These findings are relevant to ongoing national and global PPR eradication programs.

The morbidity and mortality rates during the outbreak were 50.7% and 21.6%, respectively, in sheep and 51.3% and 25.1%, respectively, in goats in the study flocks. Morbidity and mortality rates of PPR in the literature are variable depending on several factors, such as the immune status of the population, strain of the virus, species of the animal, etc. For example, a comparable morbidity rate (53% in sheep and 51% in goats) but a lower mortality rate (13.5% in sheep and 8.5% in goats) was reported from a PPR outbreak in India (Thombare & Sinha, 2009). However, a much higher average morbidity rate (75%) and mortality rate (59%) were observed in goats from PPR outbreaks in Bangladesh (Chowdhury et al., 2014).

Generally, PPR has a high morbidity rate (90%‐100%) and mortality rate (50%‐100%), especially in naive populations, but can be lower in endemic situations due to preexisting immunity. This arises from prior vaccination or virus exposure, particularly in older animals, and maternally derived immunity in young stock. Immunity in older animals is reflected in their lower mortality and morbidity in this study (OIE, 2020). Highly variable, but on average lower, morbidity and mortality rates were observed elsewhere in endemic pastoral settings in Africa (Jones et al., 2020). Although there are instances where sheep were more severely affected than goats, goats are considered the most susceptible species for PPR (Kumar et al, 2014). Similar morbidity and mortality rates were observed between sheep and goat flocks in the present study.

Economic evaluation is essential when planning control policy but is seldom done, particularly in low‐ and middle‐income countries, often due to lack of data and lack of familiarity with appropriate methods. The cost–benefit analysis of the global PPR control program considers only avoided mortality losses (Jones et al., 2016), as estimates of losses from morbidity (without death), although important, are lacking. In this study, we seek to address this gap by providing estimates of various farm‐level PPR impacts and costs and vaccination. We found that mortality accounted for approximately 75%–80% of flock‐level losses in the area studied, giving some insight into the underestimated global PPR impact resulting from the exclusion of PPR losses in addition to livestock deaths.

The household losses of ETB 7835 (USD 329.1) per affected sheep flock and ETB 7136 (USD 299.7) per affected goat flock were 14% and 13% of the annual rural Ethiopian household expenditure (proxy of household income), respectively, which was ETB 54864 (USD 2304) for 2018 (PDC, 2018). For some households, the impact would be more severe; for example, for the upper quartile sheep flocks that experience losses greater than ETB 10275 (USD 431), the household income loss would be greater than 19%. This indicates that PPR outbreaks have a large impact on the income of affected Ethiopian small ruminant keepers. Roughly comparable losses due to PPR outbreaks have been reported elsewhere in Africa. In southern Tanzania, the PPR economic impact was estimated up to a loss of 155 Euro (USD 200) per household per year (Idoga et al., 2020), and in arid and semiarid parts of northern Kenya, 21%−99% of livestock‐derived income was reported to be lost due to a PPR outbreak (FAO, 2010).

Disaggregated analysis revealed that among different components of losses, mortality accounted for 83% in goat flocks and 74% in sheep flocks. Similarly, mortality accounted for up to 88% of economic losses in a study performed in the Turkana region of Kenya (Kihu et al., 2015). The second major loss was due to weight loss, which was particularly significant in sheep flocks, accounting for approximately 23% of all economic losses. The contributions of abortion loss, treatment cost, and opportunity cost to farmers’ labour were relatively small.

This study only considered short‐term direct farm‐level impacts, and some estimates were crude (e.g., weight loss and abortion loss). The impacts of the disease associated with poor growth and reproduction performance are less sudden than mortality but may have large long‐term effects on the herd. The impact on trade and milk production (where important) should also not be overlooked.

The present outbreak impact estimates are for an endemic situation where the population had a certain level of immunity, which resulted in lower morbidity and mortality than expected from a PPR outbreak in a naive population. Finally, it is also worth noting that the morbidity and mortality data used for the economic loss estimation were based on farmers’ diagnosis of cases in their flocks. Although the outbreak investigated in this study was confirmed to be PPR, it is possible that farmers could miss or misdiagnose cases or conversely have an exaggerated memory of the outbreak, and this will have implications for the accuracy of the loss estimates. Relatedly, mixed flocks, which had larger flock sizes, were excluded from the analysis, as the farmers’ recall of the morbidity status of 100 animals in their flock was deemed unreliable. This exclusion might introduce some undefined bias in the representativeness of the flocks in the study area.

The cost per dose for a vaccinated animal was estimated to be ETB 3.00 (USD 0.13). This was lower than a previous study in Ethiopia that reported ETB 6 for a similar production system (Lyons et al., 2019). The difference could be due to larger flock sizes in our study leading to lower per‐dose vaccine delivery costs (Tago et al., 2017). Personnel cost was the major component of vaccination cost followed by transport. In a study in Senegal, personnel costs were the major cost component when the number of animals vaccinated per day was small in a smallholder mixed crop‐livestock system (Tago et al., 2017). Therefore, careful planning to mobilize smallholders to arrange their animals for vaccination will increase efficiency and reduce the vaccination cost per animal. Furthermore, the estimate of vaccination costs in our study did not include fixed costs, such as the salaries of veterinary personnel and cold chain facilities, which are shared with other veterinary services; the proportion of these costs attributable to PPR control is thought to be too small to significantly affect the estimates and conclusions of this study.

The most effective way to control PPR is a focused area‐wide mass immunization of small ruminants, as it is difficult to implement strict sanitary control measures, and stamping out in smallholder systems in countries such as Ethiopia is not feasible. However, the economic efficiency of vaccination needs to be evaluated and optimized. In the present study, although it was not possible to perform a full cost–benefit analysis of PPR vaccination, based on the high estimated impact of an outbreak and the low cost of vaccination, PPR vaccination would be expected to deliver positive economic returns.

Considering goats, where the cost per animal in the affected kebele was ETB 306, and 25% of the kebeles in the district were affected, the expected impact per animal in the district would be ETB 76.5 (306 × 0.25), approximately 13 times the cost of giving two doses of vaccination to an animal each year. This suggests that a district‐level vaccination break‐even point would be if a PPR outbreak would occur once every 13 years without vaccination, which is the case for much of Ethiopia (Fentie et al., 2018). At this point, vaccination becomes cost‐neutral for the district and profitable if outbreaks are more frequent. Even if outbreaks are less frequent, it may still be advisable to vaccinate regularly to safeguard against the massive short‐term economic shock of experiencing an outbreak and its impact on household well‐being. Furthermore, there are also the long‐term population benefits of maintaining high levels of vaccine‐derived immunity to reduce virus circulation and disease risk, preventing further spread of PPRV to new areas with progress towards eradication.

## 5 CONCLUSIONS

This study identified a high morbidity rate (51% in both sheep and goats) and mortality (22% in sheep and 25% in goats) during a PPR outbreak in small ruminants in Ethiopia. Despite this study not capturing the full impact of the outbreak, we found that affected flocks experienced significant losses, with a mean household loss of USD 329 for sheep and USD 300 for goat flocks, equivalent to on average 14% of annual income for smallholders in these systems, with many more severely affected. Three‐quarters of the economic losses were attributed to small ruminant mortality, followed by weight loss in the surviving animals.

A relatively efficient vaccine delivery system resulting from relatively large flock sizes helped keep vaccination costs low (USD 0.13 per dose, including vaccine and delivery costs). Regular vaccination against PPR in the district is likely to be economically profitable at the herd level, with additional benefits at the population level. Vaccination against PPR should be strengthened and expanded. Vaccination delivery should be planned and coordinated with local communities to increase the number of animals vaccinated per day, to maximize coverage, and to minimize vaccine delivery costs.

## CONFLICT OF INTEREST

The authors declare no conflict of interest.

## ETHICS STATMENT

The authors confrim that the ethical polcies of the journal, as noted on the journal's author guildlines page, have been adhered to and ethics approval was obtained from Institutional Review Board of UNivesity of Gondar.

## Data Availability

The data can be accessed upon request from the first author.

## APPENDIX 1

### PPR OUTBREAK IMPACT ASSESSMENT QUESTIONNAIRE

I. Study location and interviewee
  1. District           kebele            specific name of place/*Gote*
  2. Total No. of shoats in the kebele         Sheep        Goat        Total       
  4. Geo‐reference of kebele Long       Latit        Altit       
  5. Owner's name (optional)        sex     
  6. Number of livestock‐owned Cattle       sheep       goats       
II. Economic impact questions
  1. Have you ever heard of PPR? Yes/No
  2. If yes, can you describe its symptoms (Continue with questionnaire if one or more of the following signs are described?)
    A. Nasal, oral, and ocular discharges and contagious.
    B. Diarrhoea and nasal discharge and contagious
    C. Mouth lesions and diarrhoea that is contagious.
    D. Difficult breathing, diarrhoea, and contagious
  3. Have you heard of the occurrence of a PPR outbreak in your kebele in the last 3 months?
    Yes     No
  5. Did the recent PPR outbreak affect your herd? Yes No
  6. If yes, would you please provide the following information about the morbidly and mortality of the outbreak in you small ruminants herd.
    **Table:**
    **Species**	**Category**	**No Owned**	**No. of affected**	**No. died**	**No. aborted**	**No. treated**
    Sheep	Adult nonpregnant female				–	
    	Adult pregnant female					
    	Adult male					
    	Young female and male (3‐12 months inclusive)				–	
    	Lamb female and male (below 3 months) female				–	
    Goat	Adult nonpregnant female				–	
    	Adult pregnant female				–	
    	Adult male				–	
    	Young female and male (3‐12 months inclusive)				–	
    	kid female and male (below 3 months)					
  7. How much is the cost of treating animals during the outbreak (in Birr)?
  8. How many of the affected animals were treated?
